# Neighborhood Variation of Sustainable Urban Morphological Characteristics

**DOI:** 10.3390/ijerph15030465

**Published:** 2018-03-07

**Authors:** Poh-Chin Lai, Si Chen, Chien-Tat Low, Ester Cerin, Robert Stimson, Pui Yun Paulina Wong

**Affiliations:** 1 Department of Geography, University of Hong Kong, Hong Kong, China; pclai@hku.hk (P.-C.L.); chientat@hku.hk (C.-T.L.); 2 Department of Environment, Technological and Higher Education Institute of Hong Kong, Hong Kong, China; 3 Institute for Health and Ageing, Australian Catholic University, Melbourne, VIC 3000, Australia; ecerin@hku.hk; 4 School of Geography, University of Melbourne, Parkville, VIC 3010, Australia; rstimson@unimelb.edu.au; 5 School of Geography, Planning and Environmental Management, University of Queensland, Brisbane St Lucia, QLD 4072, Australia; 6 Science Unit, Lingnan University, Hong Kong, China; paulinawong@ln.edu.hk

**Keywords:** urban sustainability, morphology, road networks, green space, building volume, land use mix

## Abstract

Compact cities and their urban forms have implications on sustainable city development because of high density urban settlement, increased accessibility, and a balanced land use mix. This paper uses quantitative means of understanding urban morphological characteristics with reference to the differing qualities of the urban form (i.e., street patterns, building volumes, land uses and greenery). The results, based on 89 neighborhood communities of Hong Kong, show varying degrees of regional differences in the urban built form supported by numerical statistics and graphical illustrations. This paper offers empirical evidence on some morphological characteristics that can be estimated objectively using modern geospatial technologies and applied universally to inform urban planning. However, more studies linking these quantifiable measures of the physical form with sustainable urban living are needed to account for human comfort in the totality of environmental, social, and economic responsibilities.

## 1. Introduction 

Urban morphology describes the physical form of a city in terms of its building layout, road patterns, land uses, and green space [1]. The current challenge to urban studies is the very rapid large-scale urbanization in non-Western countries [2]. Given that more than 60% of the World’s increase in urban population is occurring in Asian cities that are pursuing high-rise and compact urban development [3] a better understanding of the physical living environment and urban quality is necessary to deal with the many facets of urbanism in Asian cities. Studies have shown that the urban form of a city can affect air dispersion [4,5,6], induce urban heat islands [7,8], cause health and thermal comfort problems [9,10,11], and influence energy consumption [12]. Morphological differences within a built environment exist because of land use patterns and the varied neighborhood preferences of certain social groups [13]. Also, urban land uses containing tall buildings have significantly different and greater variation of morphological qualities compared to other urban land uses [14]. 

A wide range of descriptive and quantitative urban morphological measures have been developed over the years [15,16,17]. There are many causes for the lack of consensus in the measures. Firstly, classic urban form studies have focused on specific geographic regions (mostly in western countries) and constrained by urban form traditions. Secondly, changes to the patterns of recent city forms have been dramatic. Thirdly, the structure of urban form is the product of social/cultural processes and there are different kinds of structure with different characteristics at different geographic scales [4]. 

Urban morphology can affect the quality of urban life and pleasantness to live. Overcrowding, congestion, air pollution, and lack of green space are some of the negative attributes of urban development. There is, however, an increasing focus in linking urban form and sustainable city development. These efforts can be summarised into two competing models of sustainable urban form: (1) compact cities based on ecological footprints; and (2) green cities based on more open type of urban structure [18]. Urban development in Asia tends towards the first model and its sustainability has been broadly examined from two key aspects of physical and socio-cultural contexts. More sustainable physical aspects include compact urban form, mixed land use, walkable environment, and more green or open spaces [19,20] whereas sustainable socio-cultural settings entail green jobs, clean energy, environmental protection, safety, sewage treatment, and waste reduction [21,22,23,24]. The physical constructs of sustainability can be studied using the urban morphological methodology, which is illustrated in this paper.

Urban compactness is often perceived through high population density, mixed-use, and efficient transport [25,26]. Analyses of urban form today are related to building density and building height or the built-up volume to the third dimension [27]. With geospatial technologies, building typologies and the analysis of patterns of urban structure in recent years are increasingly based on geometric characteristics such as the shape of the buildings and their distance separation. Studies have also shown significant differences in building density and structures among urban districts constructed in different time periods [28]. For example, building height and density have increased in many cities with adverse effects on the provision of daylight [6,29], wind speed and strengths [30]), and green space [31]. However, dense and concentrated vertical housing is most favorable from an environmental point of view in reducing the ecological footprint of a household [18]. 

Land use planning may affect human behaviour. It has been proven that more varied land use patterns encourage walking or biking while reducing driving [32,33,34]. This is to say, a diversified land use structure can promote more physical activities and less dependence on cars. Because each land use type is catered for a different function, a neighborhood with more diversified land uses can fulfil various demands more easily. There is, however, the need to consider land use compatibility amid diversity. Past research has considered five common land use types of residential, commercial, industrial, government, and open area wherein the industrial land use type is regarded incompatible with the others [34,35]. Indeed, industrial lands have a lower land rent [36] as their presence is often associated with negative impacts of poor air quality or higher crime [37,38]. In a vertical city such as Hong Kong, the mixed land use type is quite common because a building complex can have multiple functions (e.g., the lower levels for retail and commercial purposes but upper floors for residential use).

The physical layout of an area, including the size and designated land uses, is often shaped by street typology which affects walkability and accessibility. Street patterns or the measurement of geometric accessibility at the street-building level of representation have evident usefulness for urban analysis [39,40,41]. Higher intersection densities can increase connectivity and offer greater variety of potential routes [34]. Road safety in terms of fatal vehicle crashes also improves with denser street networks with higher intersection counts per area [42,43]. Both the extent of road networks (intersection and overall length) and building footprints exhibit varying degrees of negative association with the extent and quality of green space [31]. Greenery and vegetation cover can alleviate the adverse effects of increasing urban expansion and densification [31,44]. Urban green spaces in most cases include public parks, open spaces (covering sports fields; derelict land; edges of roads, railways and waterways; private gardens; and remnant patches of natural vegetation), as well as individual street trees. 

Hong Kong has a compact urban built form consisting of high-rise and high density dwellings and mixed land uses. This high density high-rise built form is considered by some researchers to be a model of sustainable transport development but Hong Kong has also been criticized for its severe lack of green and open spaces for quality living conditions [45]. This paper examines the implications of urban forms in 89 neighborhood communities of Hong Kong with reference to the effects of varying qualities of street patterns, greenery, building volumes, and land uses. It hopes to establish a link between quality of urban life and environmental sustainability at the neighborhood level in terms of objective sustainability indicators referred by Marans [46]. It also attempts to examine quality of life experienced across a range of environmental and morphological dimensions that relate to 89 neighborhood communities located in both urban and urban-rural fringe, with particular focus on transport infrastructures that are strongly linked to planning [47]. The visualization tools described in this article can also serve as an initiative to a broader and deeper exploration of the multi-dimensional realm of sustainable urban morphological characteristics.

## 2. Methods

### 2.1. Study Area

**Figure 1:**
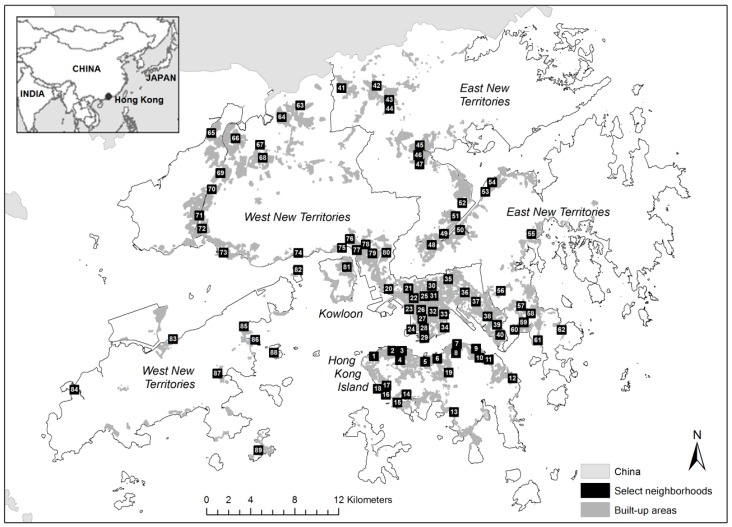
Geographic distribution of 89 neighborhoods of Hong Kong.

Hong Kong lies in the southern coast of China beneath the Pearl River Delta. Located between latitudes 22°08′ N and 22°35′ N and longitudes 113°49′ E and 114°31′ E, it has more than 200 islands and occupies a total land area of 1104.41 km^2^ [45]. Its major geographic regions include the Hong Kong Island (HKI), Kowloon Peninsula (KLN), East New Territories (ENT), and West New Territories (WNT) (Figure 1). Urban development in Hong Kong is highly clustered with roughly 24% of its total area allotted for residential, commercial, industrial, transportation, and other urban activities [48]. Around 40% of land area in Hong Kong is still covered by country parks, most of which is mountainous. With over 7 million population in 2016 [49], Hong Kong is one of the most densely populated cities in the world which explains its trend towards vertical development. 

Because of historical development and new town planning initiatives, the morphological characteristics of new towns in the urban-rural fringe (i.e., New Territories) are significantly different from urban development in earlier periods. Recent studies have shown that the environmental performance of neighborhoods is important to the health and well-being of their residents [50]. As many Asian cities are undergoing urbanization at unprecedented speed since 2000, high-density urban development is inevitable. Examining distinctive features of the environmental performance of neighborhoods in an ultra-dense metropolis like Hong Kong afford us a means to rethink ‘sustainability vs. livability’ implications in future high-density environments.

The study identified a total of 89 neighborhoods representing different degrees of urbanization (Figure 1). The spatial unit of a neighborhood is fixed at 800 m × 800 m square area (see also [51,52]) for consistent and meaningful comparison. These neighborhoods were selected using a two-step process: (i) obtaining official place names and their geographic locations established by the Planning Department; and (ii) ensuring each neighborhood site covers important residential estates based on the GeoInfo Map [53] (The spatial coverage of the 89 neighborhoods is well spread even though their locations are unevenly distributed among the four geographic regions: HKI (19), KLN (21), ENT (22), and WNT (27).

### 2.2. Data

The study made use of official data from the Survey and Mapping Office of the Lands Department of Hong Kong, including digital topographic map series (B5000 and B10000), digital orthophotos (DOP 5000), building data (BG1000), and road centerline files (RG1000) for 2011. Other data for cross-validation comprised SPOT-5 panchromatic image of 2.5-m resolution and multispectral image of 10-m resolution for the year 2010, as well as the outline zoning map series (OZP, 2006–2010) from the Hong Kong Planning Department. These data were used to extract various urban morphological metrics described below. 

### 2.3. Morphological Measures

Urban morphological metrics selected for the study should describe spatial characteristics based on size, composition, and intensification of the built-up area [54]. The five selected metrics included the number of road intersections, road density, building volume, percent greenery, and compatible land use mix (CLUM) index. The land use mix or LUM index [55] is a measure to represent the degree of diversified land uses within an area. LUM values range between 0 and +1, with “0” indicating homogeneity (i.e., the presence of a single land use type where there is no mixed use) and “1” showing heterogeneity (i.e., the presence of all land use types at equal proportion). In general, LUM scores approaching “1” indicate better environmental sustainability in terms of its potential to engage its residents in physical activity for better health [33]. These measures were estimated by means of spatial analysis methods provided within modern geographic information systems (GIS) and related software products. This study employed ArcGIS v.10.2.1 [56].

The number of road intersection and road density are useful measures to describe urban typology [57,58]. The number of intersection within a neighborhood was extracted from the RG1000 road centerline file using a GIS. Road density is defined as the percentage of total road surface out of the total land area (i.e., 800 m × 800 m minus water area, if any). Road surface area was computed from the B10000 land polygon layer covering all main and secondary roads, flyovers, tracks and bicycle tracks except express roads (because express roads are for intercity instead of intracity travel). 

Building volume within a neighborhood is a measure of building densification. It was estimated by summing products of all building footprints and their corresponding building heights. Ground floor building footprints were retrieved from the B5000 building feature layer while building heights were obtained from the BG1000 building data.

A land use map is an important layer for understanding the relationships between developed and natural areas. Hong Kong has seven major designated land uses: commercial, greenery, industrial, institutional, mixed, open area, and residential. The greenery classification includes the following uses: agriculture, conservation, grassland, country and urban parks, greenbelt, and recreation. Since digital land use maps of Hong Kong are not readily available, the layer was derived by combining data from multiple sources, including B5000, SPOT-5 images, and OZP. The percentage of each type of land uses within an 800 m × 800 m neighborhood was processed using GIS-based functions. The *LUM* index for each neighborhood was calculated using the formula expressed below:

$$LUM=-\sum_{k}\left(p_{k}\ln p_{k}\right)/\ln N$$

where *N* is the number of compatible land use types; *k* is the type of land use; and *p* is the proportion of a specific type of land use within a neighborhood.

To address the issue of land use compatibility amid diversity, the study excluded industrial land use in the computation of LUM scores because of its negative effects, such as poor air quality or higher crime [37,38]. In addition, areas with mixed land use designation were allotted in equal proportions to commercial and residential land use types. 

### 2.4. Methods of Analysis

Five morphological metrics were computed for each neighborhood. Each metric recorded in different measurement units was converted into standardized scores to allow for fair comparison. The unity-based normalization process, as shown below, was applied to bring all values into the range (0, 100):

$$X^{\prime }=\left(\frac{X-X_{\min}}{X_{\max}-X_{\min}}\right)100$$

where X is the selected variable; $X^{\prime }$ is the normalized or scaled score of the variable; and $X_{\max}$ and $X_{\min}$ are the maximum and minimum values respectively.

**Figure 2:**
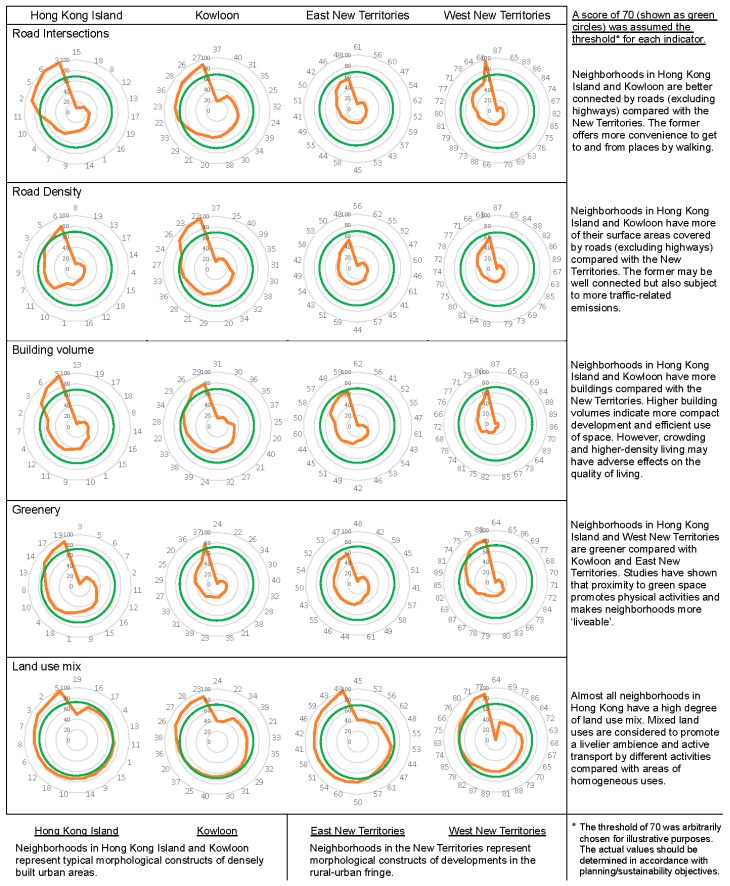
Radar charts of morphological metrics (based on normalized scores) by geographic regions of Hong Kong.

**Figure 3:**
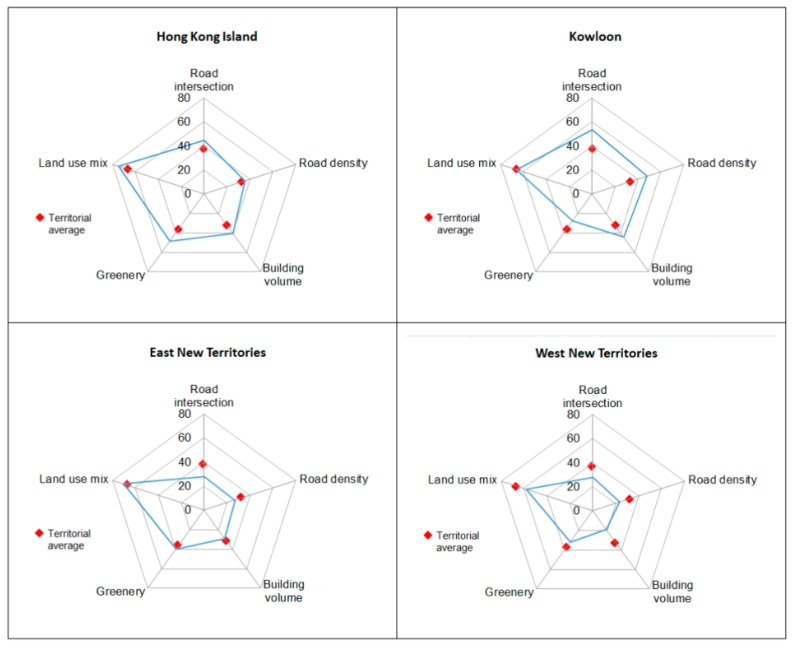
Summary radar charts of morphological metrics (based on mean normalized scores) by geographic regions of Hong Kong.

The standardized metric scores were sorted and grouped by four geographic regions of Hong Kong. Although textual or tabular presentation is excellent in conveying exact values, they are less effective in communicating an overall impression and for comparative analysis [59]. This study made use of the radar chart graphical representation. A radar chart is also known as a spider chart. The number of vertices along the outer edge of a radar chart can vary according to the number of observed cases or members [60]. Each axis is independently scaled to be between 0 and 100 by normalizing data on the basis of its biggest value. A radar chart can display the relative values of multi-dimensional data by shape for visual examination and its standardized format also facilitates quantitative comparison [61,62,63]. In this study, the 89 neighborhoods were sorted into four geographic regions and a radar chart of a chosen variable was graphed for each geographic region to examine regional differences by comparing the size and shape of the polygons, see Figure 2, and box plots. A box plot or box and whisker diagram shows the range, median, and other characteristics of responses (such as shape, center, spread, unusual features) for a group of numerical data [64]. It enables visualization of the distributional characteristics of the ordered scores by indicating data spread, labelling the division lines of quartiles (25%, 50% or median, and 75%), and highlighting extreme or outlier values. Placing box plots of standardized scores against each other can reveal data variability (i.e., clustering or uniform distribution, see Figure 3) supplemented by statistical analysis to examine the results. All data processing and analysis procedures were carried out using Microsoft Excel (Microsoft Corporation, Redmond, WA, USA) and SPSS ver. 24 (IBM Corporation, Armonk, NY, USA). 

## 3. Results

### 3.1. Numerical Analyses

Table 1 shows the mean normalized scores of the five morphological metrics by geographic regions of Hong Kong. It shows that KLN reported very high mean scores for 3 of the 5 morphological metrics with very dense and well connected road network, very high building volume, reasonably mixed land use, and a shortage of green space. HKI is characterized by dense road network, high building volume, very mixed land use and quite green. In reference to territorial averages (mean values of morphological metrics for all 89 neighborhoods), HKI and KLN reported above average scores for road intersection, road density, and building volume. These three metrics reveal that HKI and KLN are more compact than the New Territories in their urban built form. Table 1 also shows that KLN is exceptionally low in green space whereas neighborhoods in Hong Kong tend towards mixed use urban forms.

One-way analysis of variance (ANOVA) was applied to determine whether the mean normalized scores are statistically significantly different from each other. Table 2 shows that there are statistically significant differences (*p* ≤ 0.05) between the means of all five morphological metrics. Post hoc analysis using various tests (i.e., Tukey HSD, Scheffe, and Bonferroni) was used to conduct other than just pairwise comparisons to determine which specific groups differed from each other for each morphological metric (not shown here). The results found that neighborhoods within the four geographic regions (HKI, KLN, ENT, and WNT) exhibit different morphological characteristics based on the five metrics. The results also indicate varying degrees of regional differences in the urban built form.

### 3.2. Graphical Analyses

Figure 2 shows the radar charts based on normalized scores of the five morphological metrics for all 89 neighborhoods arranged by four geographic regions. The radar charts show not only regional variation of morphological characteristics but also compositional differences within each region. Assuming an arbitrary score of 70 as the sustainability threshold for each of the morphological metrics (indicated as green circles in Figure 2), it can be seen that some neighborhoods have exceeded the suggested development threshold. Exceeding the threshold may bring negative impacts, as in the case of “Road Density” where residents of these neighborhoods are subject to more traffic-related emissions. Figure 3 displays another set of radar charts to show the mean normalized scores of five morphological metrics by distinct geographic regions. The territorial averages were also plotted on each radar chart to enable visual comparison of the overall performance within each region. The results show HKI and KLN are more similar based on mean normalized scores of the five morphological metrics compared with ENT and WNT that look more alike. The radar charts also show subtle morphological differences among the geographic regions. 

**Figure 4:**
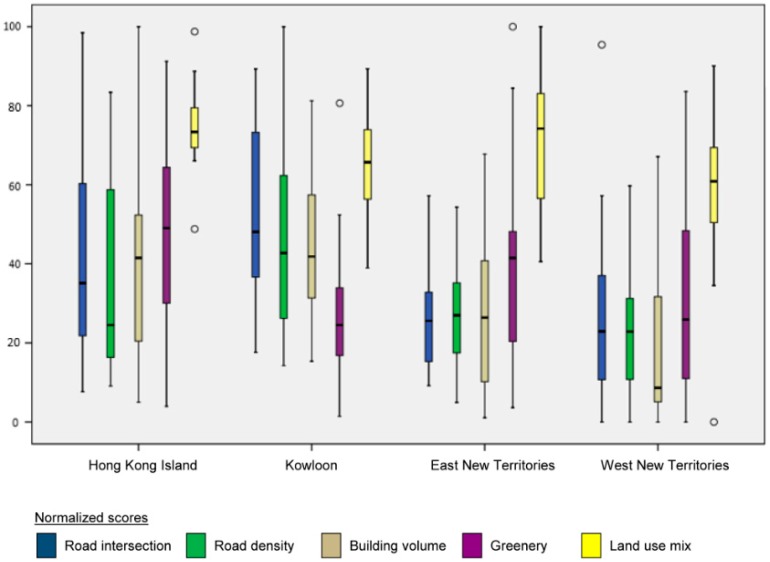
Box plots of morphological metrics (based on normalized scores) by geographic regions of Hong Kong.

Figure 4 displays box plots of all morphological metrics by geographic regions. It is evident that LUM exhibits uncharacteristically high scores and clustered distribution compared with the other metrics. This observation signifies that most neighborhoods in Hong Kong have mixed land uses. In general, normalized scores for four of five morphological metrics (i.e., with the exception of LUM) for both HKI and KLN are more widespread, indicating large within group variation in neighborhood morphological characteristics for the two regions. Normalized scores of the first three metrics (excluding greenery and LUM) for ENT and WNT are distributed toward the lower end. This finding reflects that the urban built form, comprising of roads and buildings, of neighborhoods in the New Territories are less dense.

## 4. Discussion

The study examines physical constructs of urban sustainability based on morphology. Although sustainability is a complex issue involving multiple concerns, the physical form of cities including the way people live in them and travel around them constitutes an important element of consideration. The compact city arguments have focused on increasing the density of urban development, improving accessibility, encouraging mixed land uses, and enhancing green space. These components can be assessed in an objective manner using modern geospatial technologies but they must be considered in light of the limits to growth. The study made use of neighborhoods located in the core and peripheral areas of Hong Kong to examine their morphological qualities based on five metrics—road intersection, road density, building volume, greenery, and LUM. These metrics do overlap, to some degree, and it is difficult to completely isolate individual factors. For example, the extent of the road network and the building footprint in an area are roughly equal in their negative contribution to the extent of green space.

Despite apparent simplicity of the morphological metrics, the study demonstrates the feasibility of deriving these measures and making sense of the quantitative results. Descriptive statistics (Table 1) are useful for summarizing basic features of the morphological metrics while ANOVA results (Table 2) are good for investigating within and between group differences for statistical significance. Although numerical and statistical analyses can afford a means to quantity strength of evidence, they are not always easy to interpret. Numerical and graphical analyses complement each other. Here, radar charts (Figure 2 and Figure 3) were drawn to provide graphical comparison and overview of the morphological metrics based on size and geometry. Size allows for visual comparison of the magnitudes of selected morphological quality within a given region. The geometrical properties (such as angle of connection and shape) can inform similar observations and identify outliers. Furthermore, radar charts similar to those in Figure 3 can be created for each neighborhood to identify coherent neighborhood morphology.

The box plots (Figure 4) enable comparative assessment of the distributional patterns of morphological characteristics by indicating data spread and variability (i.e., whether data distribution is clustered or uniform). Similar to post hoc analysis in one-way ANOVA, the box plots provide visual assessment about which specific groups differ from each other for each morphological metric. Indeed, box plots deliver not only more consolidated view of the data spread but also an effective way for cross comparison by visual inspection. 

Cities are ranked or rated by many ways, such as population, gross domestic product, quality of life, cost of living, and crime. The visualization tools described in this paper serve as an initiative to a broader and deeper exploration in the multidimensional realm of sustainable urban forms. Although a myriad of indicators do exist to compare performance between urban areas, visualization makes comparing cities with various attributes easy and straightforward [65] even though more time is needed to comprehend a radar chart than traditional curve-line graphs. Indeed, city-based indices ignore spatial variability within a city. This paper uses 800 m × 800 m areas as spatial units and normalized measures to explore intracity and regional differences in a consistent and systematic manner. 

A major limitation of the urban morphological methodology concerns a lack of research in the applicability thresholds for best practice in sustainable development. This study assumes that increasing values in morphological metrics will enhance urban sustainability. That is to say increasing density, improved accessibility, intense LUM, and more green space have positive impacts on urban sustainability. However, the various morphological metrics have negative influences on each other within a unit of space. For example, increasing built density will take away lands for green space, as illustrated by KLN in this study. Further investigation is thus needed to establish thresholds of having the maximum expected utility of different morphological metrics in urban development. In particular, how much green space is needed to mitigate the effects of building at high density? Noting that the issue of urban sustainability is more than the physical form, future study should consider also environmental concerns, social acceptability, and economic viability.

## 5. Conclusions

It is important to have quantitative criteria for sustainable urban development that can be applied in other cities. The study introduces five morphological characteristics that can be derived objectively from aerial images of a city. There remain, however, many questions about urban sustainability. Are more compact, higher density, and mixed urban forms more environmentally sound and more efficient for transport? How much urban green space do people need in their neighborhood to be healthy and happy? With increasingly intense debates in policy and practice about sustainability, this paper offers partial insight on some morphological metrics that can be applied universally and used to inform policy planning. It provides food for thought on quantifiable measures that can be used as evidence for urban planners, designers, as well as decision and policy makers to support their proposals towards more sustainable urban living. 

## Figures and Tables

**Table 1:** Mean normalized scores of morphological metrics by geographic regions.

Morphological Metrics	Mean (89)	Std. Dev.	Std. Error	95% CI (Mean)		Mean Scores by Regions			
				Lower Bound	Upper Bound	HKI (19)	KLN (21)	ENT (22)	WNT (27)
Road intersection	37.29	24.88	2.64	32.05	42.53	44.63	53.16 *	27.63	27.65
Road density	32.68	21.62	2.30	28.12	37.23	35.81	48.02 *	26.74	23.38
Building volume	32.01	23.53	2.49	27.05	36.97	40.77	44.21 *	29.12	18.72
Greenery	36.28	24.71	2.62	31.08	41.48	48.67 *	27.18	39.96	31.65
Land use mix	66.56	16.83	1.78	63.01	70.10	74.76 *	66.16	70.53	57.87

CI = confidence interval; HKI = Hong Kong Island; KLN = Kowloon; ENT = East New Territories; WNT = West New Territories. * Maximum mean score for each morphological metric.

**Table 2:** Results of one-way analysis of variance (ANOVA) on mean normalized scores.

Source of Variation		Sum of Squares	*df*	Mean Square	*F*	*Sig.*
Road intersection	Between Groups	10,876.28	3	3625.43	7.070	0.000 *
	Within Groups	43,586.84	85	512.79		
	Total	54,463.13	88			
Road density	Between Groups	8237.40	3	2745.80	7.097	0.000 *
	Within Groups	32,883.82	85	386.87		
	Total	41,121.22	88			
Building volume	Between Groups	9534.75	3	3178.25	6.895	0.000 *
	Within Groups	39,181.04	85	460.95		
	Total	48,715.78	88			
Greenery	Between Groups	5534.23	3	1844.74	3.255	0.026 *
	Within Groups	48,179.90	85	566.82		
	Total	53,714.13	88			
Land use mix	Between Groups	3666.29	3	1222.10	4.889	0.003 *
	Within Groups	21,248.39	85	249.98		
	Total	24,914.68	88			

* Significant at 5% level of significance; *df*: degrees of freedom; *F*: f value; *Sig.*: significance of F.
